# Topically applied vitamin C increases the density of dermal papillae in aged human skin

**DOI:** 10.1186/1471-5945-4-13

**Published:** 2004-09-29

**Authors:** Kirsten Sauermann, Sören Jaspers, Urte Koop, Horst Wenck

**Affiliations:** 1 Research and Development, Beiersdorf AG, Hamburg, Germany

## Abstract

**Background:**

The influence of ageing on the density of the functional entities of the papillae containing nutritive capillaries, here in terms as the papillary index, and the effect of topically applied vitamin C were investigated by confocal laser scanning microscopy (CLSM) in vivo.

**Methods:**

The age dependency of the papillary index was determined by CLSM on 3 different age groups. Additionally, we determined the effect of a topical cream containing 3% vitamin C against the vehicle alone using daily applications for four months on the volar forearm of 33 women.

**Results:**

There were significant decreases in the papillary index showing a clear dependency on age. Topical vitamin C resulted in a significant increase of the density of dermal papillae from 4 weeks onward compared to its vehicle. Reproducibility was determined in repeated studies.

**Conclusions:**

Vitamin C has the potential to enhance the density of dermal papillae, perhaps through the mechanism of angiogenesis. Topical vitamin C may have therapeutical effects for partial corrections of the regressive structural changes associated with the aging process.

## Background

A consistent feature of aged and photoaged skin is the flattening of the epidermal-dermal junction, evidenced in histological sections as a loss of rete ridges and the disappearance of papillary projections. The depth of interdigitation of the retepegs and the dermal papillae decreases with age [1]. Using confocal scanning microscopy sections horizontal to the surface can be obtained optically in contrast to conventional transverse sections which are perpendicular to the surface. Comparison of confocal images with corresponding histological sections have been made by the group of Gonzales to validate the method [2-5]. In confocal images the dermal papillae appear as dark circles surrounded by bright reflecting rings of basal cells containing highly reflective pigment [6]. Each papilla contains a single nutritive capillary loop [7]. The density of these functional entities of dermal papillae containing a single nutritive capillary loop can be evaluated more accurately and precisely by confocal microscopy than by conventional histology. This is partly due to the avoidance of shrinkage artefacts after fixation for histological sectioning. Confocal microscopy is more suitable to construct a three dimensional image than conventional serial sections are [8].

In extremely aged skin, papillae virtually disappear and the junction with the atrophic epidermis is a straight line versus undulations in younger skin. The papillary dermis also thins along with a loss of capillaries. Grove described that the corneocytes in aged skin become larger as a result of decreased epidermal turn-over [9]. Similarly, cells of the granular layer become larger, indicating a slow epidermal turn-over in aged skin [6].

Unlike for example in mice, in humans like in primates in general and in guinea pigs vitamin C must be supplied exogenously in the diet. Diets deficient in vitamin C cause the multiple clinical signs of scurvy.

Stones evolutionary treatise showed that a genetic mutation resulted in the loss of the ability of humans and some other animal species to synthesize vitamin C [10]. The main mechanism for the symptoms of scurvy seems to be the instability of non-hydroxylated forms of collagen, as vitamin C is needed for the hydroxylation of prolin [11]. There is evidence that topical vitamin C might be beneficial in several unrelated conditions. Topical vitamin C has been reported to improve wound-healing [12]. Roshchupkin has shown that topical vitamin C is protective against immediate effects of ultra-violet radiation on human skin leading to an increase in the dose required to induce erythema [13]. Topical vitamin C protects against ultra-violet-induced carcinogenesis [14]. The level of vitamin C in the skin decreases with age, especially in the epidermis [15,16]. Topical vitamin C increases the mRNA levels of collagens I and III, and their processing enzymes in humans [17,18]. Humbert showed the potential of ascorbic acid to improve the clinical appearance of photoaged skin and to reduce facial wrinkles [19,20].

Retinoids, too, are known to improve the clinical appearance of photoaged skin and to promote the downgrowth of rete ridges, restoring the undulating dermo-epidermal interface [21]. A functional dermal-epidermal junction provides a better protection against mechanical stress which might detach the epidermis and lead to erosions [22]. The increased availability of oxygen and nutrients should also enhance repair after wounding and reverse the structural changes associated with photoaging.

The height of the epidermal-dermal junction like it is measured as a parameter in conventional histology, can be determined by confocal microscopy only in a very inaccurate manner because of the horizontal orientation of the images. The aim of this study is first to establish a CLSM measurable parameter comparable to the height of the epidermal-dermal interface. It should be sensitive to find age-associated changes of the dermal-epidermal interdigitation. Second to examine the effects of a topical vitamin C containing moisturizing cream on the developed CLSM parameters. We used a dose of 3% vitamin C as it shows a good stability and a good release of the active ingredient in a special emulsion system [23].

## Methods

In the first experiment (I), three groups of volunteers of different ages were compared measuring the density of the functional entities consisting of a dermal papilla and the nutritive capillary (papillary index). In a second experiment (II) a topically applied cream containing 3% vitamin C and its excipient were tested on the volar forearms of 33 volunteers. The effect of vitamin C cream was compared to excipient and to untreated control sites, respectively. (III) This experiment was repeated with a slightly different vehicle and for a two month treatment period only.

### Instrument

The Vivascope 1000 (Lucid Inc., Rochester, N.Y.) is a commercially available confocal laser scanning microscope, which allows to examine human skin in vivo non-invasively. The system uses a Laser source with a wavelength of 830 nm, an illumination power up to 20 mW on the object and water immersion. In all three studies two different fields of view were used to obtain the papillary index (640 μm × 480 μm), and the projection areas of cells in the granular layer (230 μm × 88 μm). The lateral resolution of the instrument is 0.4 μm, the vertical resolution is about 1.9 μm.

### Parameters

In confocal images of the forearm the epidermal-dermal interface shows dark round areas, the dermal papillae, surrounded by bright circles of basal cells, containing melanin granules and therefore reflecting strongly. Capillary loops are located in the centre of dermal papillae as black holes, often showing bright erythrocytes flowing through the capillary. The density of dermal papillae was evaluated by counting the papillae containing a capillary loop. 20 fields of view were investigated on each test site.

The size of cells in the upper granular layer (A_gran_) was evaluated by saving images of the most apical plane of the epidermis that still showed living dark nucleated cells. Although in the same image you can have virtual sections of different cell layers, the cells belonging to this layer right underneath the stratum corneum can be identified by the characteristic morphologic features the cells display. The size of cells was analysed using the image analysis program image tool. At least 20 cells in at least three different images were analysed by trained examiners.

### Subjects and treatment

(I) In the first study three groups of female volunteers were studied: twelve, ages 18 to 25, eleven, ages 40 to 50 and twelve, ages 65 to 80 years. Informed consent was obtained. Images were obtained on the volar side of the right forearm during the winter months.

(II) In a second experiment a cream containing 3% vitamin C was compared to its vehicle on a group of initially 36 volunteers. 33 volunteers finally participated using it twice daily. Applications for four months to mid-volar forearms of healthy female postmenopausal subjects, ages 45 to 67 years (mean 55.3 years), phototype II and III. One area was left untreated. The double-blind study was conducted in winter. Informed consent was obtained. Evaluations were obtained at baseline and each month for four months, with one and two months follow-ups after stopping the treatment.

(III) To determine reproducibility a second study was conducted over an eight weeks treatment period with a slightly different vehicle. The parameter of interest was the papillary index. Informed consent was obtained. Measurements were made after 20 minutes of acclimatisation at 21°C and 50% relative humidity.

### Statistics

Morphometric results are expressed in box-whisker-plots as the median +/- the quartile. Statistical analysis used the Student t-test for normally distributed data or the U-Test (Mann-Whitney) for the age comparison study. The Wilcoxon test was used for analysing the effect of the vitamin C studies. Statistical significance was established for the 95% confidence level (p < 0.05).

## Results

**Figure 1:**
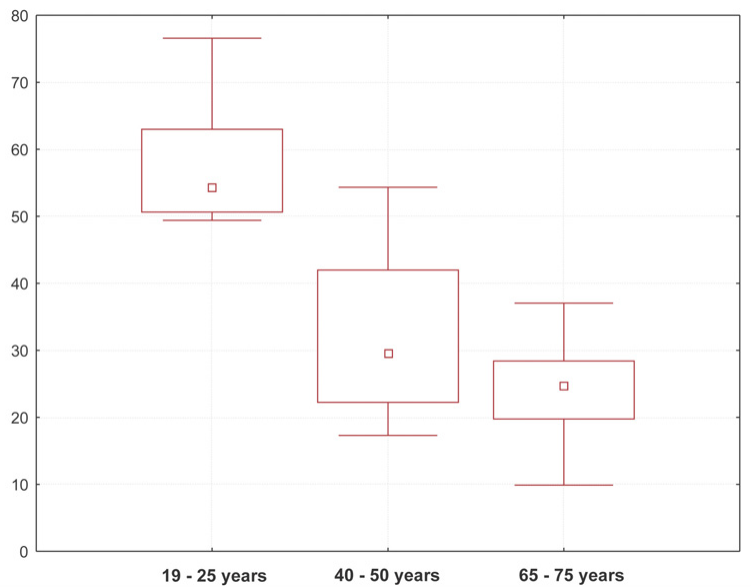
Box-plot of papillae per mm^2 ^in three age groups

(I) The papillary index in the young group, with a median of 54.3 papillae per mm^2^, was significantly the highest of the three age groups. The middle aged group showed a significantly higher index, median 29.6 papillae per mm^2^, than the oldest group, median 24.7 papillae per mm^2^. The decrease of the papillary index was more pronounced between the younger group (18 to 25 years) and the middle-aged group (40 to 50 years) than between this group and the old group (65 to 80 years) (fig. 1).

In the elderly group there were often large segments of flat epidermal-dermal junctions with a complete loss of papillae. In these regions the microvasculature consisted of horizontally orientated vessels having noticeably larger diameters than in young skin, probably venules rather than capillaries. A few curled remnants were occasionally found just beneath the atrophic epidermis.

**Figure 2:**
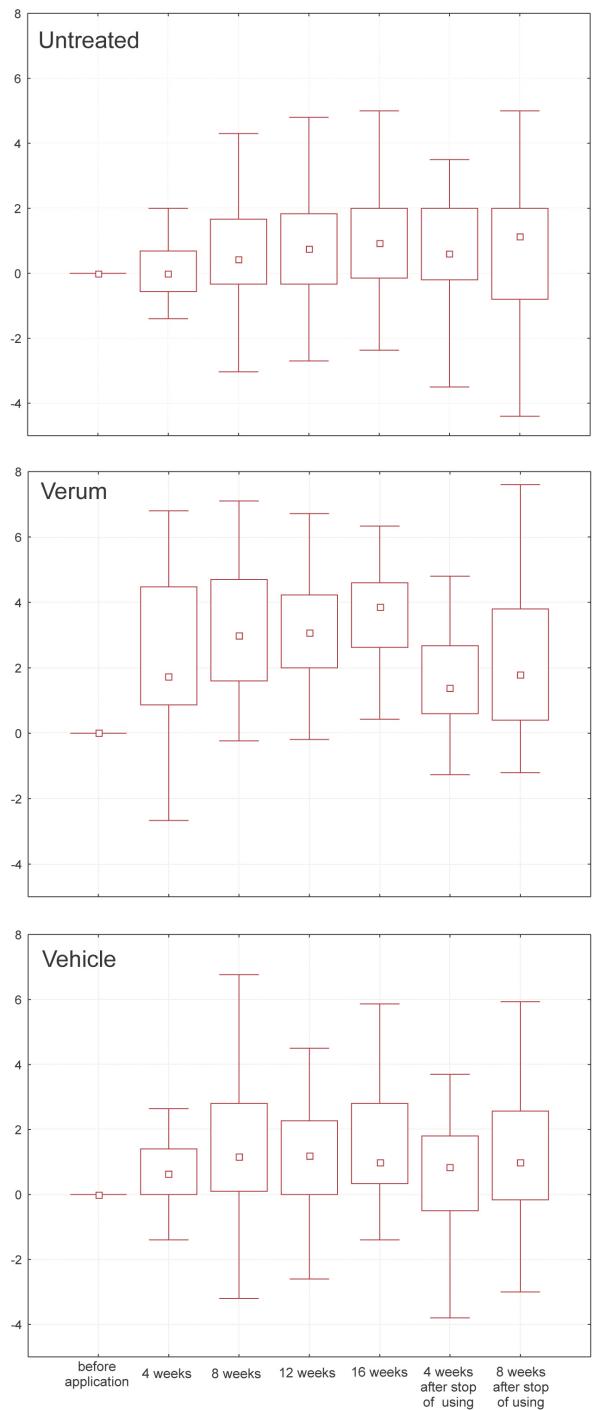
Box-whisker-plots of papillae per mm^2 ^during topical vitamin C; t0 = baseline, t1 to t4 = after 1 to 4 months, t5 and t6 = 1 and 2 months after stopping

The vessel walls could not be resolved into endothelium, pericytes or smooth muscle cells by confocal examination, owing to low reflectiveness.

(II) From four weeks onwards ascorbic acid treated areas showed a significantly higher density of capillary containing papillae than untreated and vehicle treated sites (fig. 2, p-values in table 1).

**Table 1:** statistical analysis of the verum treatment study: p-levels, Wilcoxon Matched Pairs Test

	vehicle vs verum	untreated vs verum	untreated vs vehicle
t0	58,70%	21,10%	36,40%
t1	1,40%	0,35%	95,00%
t2	0,03%	0,02%	62,60%
t3	0,02%	0,03%	97,00%
t4	0,01%	0,01%	87,30%
t5	0,60%	15,30%	28,10%
t6	4,28%	12,50%	77,30%

Statistically significant differences were also observed in the vitamin C treated sites from four weeks onward in comparison to the beginning of the study. The untreated as well as the vehicle treated site showed a slight, but significant increase (from median 25.3 to 26.9 papillae per mm^2 ^for the untreated and from median 22.7 to 30.3 papillae per mm^2 ^for the vehicle treated sites) over the treatment period as well.

The significance of the difference between the verum and the two control sites were no longer evident two months after stopping treatment. Despite of the different median values for the areas at t0, no significant differences between these baseline values could be detected.

It is noteworthy that the newly formed papillae could not be distinguished from the preexisting ones. Neither abnormal formations of blood vessels like multiple capillaries in a papilla, parallelisation of the vessels or highly enlarged diameters of the vessels nor signs of inflammatory reactions e.g. rolling or adhesion of leukocytes to the vessel walls were observed.

**Figure 3:**
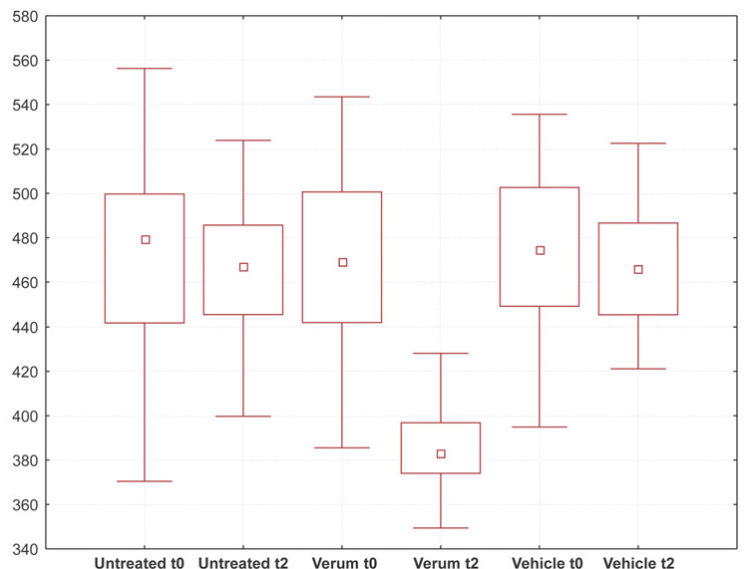
Box-whisker-plots of areas (μm^2^) of cells in the apical granular layer; t0 = baseline, t2 = after 2 months of treatment

The projection areas of the cells in the upper granular layer showed a significant decrease of about 16% from 472 μm^2 ^to 383 μm^2 ^in the vitamin C treated site, in comparison to baseline and the two control sites. Significant difference between vehicle and untreated controls compared with each other and with the initial status at the beginning of the study were not detected (fig. 3).

(III) The increase of the density of the papillae under influence of vitamin C was reproduced by repetition of the treatment schedules, ruling out the likelihood of changes due to chance.

## Discussion

The decrease of the height of the epidermal-dermal junction is a well described histological finding in aged and photoaged skin [22,24]. We conclude from our data that ageing results in a damage of the papillary dermis and of the vascular network in this layer and a decrease of the papillary density over time.

Vitamin C has been reported to improve the clinical appearance of photoaged skin and to enhance the synthesis of composite elastin fibres and of collagen [19]. Our data indicate that the topical application of vitamin C partially restores the anatomical structure of the epidermal-dermal junction in young skin and increases the number of nutritive capillary loops in the papillary dermis close to the epidermal tissue in the aged skin of postmenopausal women. The increase in the density of papillae after vitamin C treatment can not be interpreted only as a sign of epidermal hyperplasia with an enlarged area covered with basal layer and the epidermis growing down into the dermis. More likely it is linked to a restructuring of the papillary dermis, as the top of the newly formed papillae and the capillaries are localized above the average height of the plane basal layer seen predominantly in aged skin. This suggests restoration to a more normal functional state of the epidermal-dermal interdigitation and of the overlying epidermis.

In aged skin, the cells in the apical granular layer show larger projection areas than in the skin of younger individuals [8] similar to the enlargement of the size of corneocytes with age described earlier by Grove and Kligman [9]. Kligman shows an inverse relationship between turnover and the size of corneocytes [9]. A similar relationship could be shown for the size of cells in the granular layer [25]. Parallel to this, the smaller size of granular layer cells in the vitamin C treated areas can be interpreted as a sign for higher proliferative activity of the epidermis.

Another implication of the increase in papillae is that new blood vessels are formed during the treatment with vitamin C. The newly formed blood vessels show a normal anatomical structure in confocal microscopical examination and are apparently integrated in a healthy vascular architecture. In the confocal images there are no signs of pathologic changes of the vasculature like, for example, increased diameter, parallel orientation or clew of vessels in a papillae. No perivascular infiltrations of lymphocytes could be observed during the treatment period.

The mechanism, by which Vitamin C restores dermal papillae is unknown.

## Conclusions

These are early results which strongly suggest that topical vitamin C may have important anti-aging effects in correcting the structural and functional losses associated with skin aging.

## Competing intrests

The studies were funded by the Beiersdorf AG, Germany. Parts of the results presented in the manuscript are subject of patents pending by Beiersdorf AG.

## Pre-publication history

The pre-publication history for this paper can be accessed here:


